# A Self-Help App for Syrian Refugees With Posttraumatic Stress (Sanadak): Randomized Controlled Trial

**DOI:** 10.2196/24807

**Published:** 2021-01-13

**Authors:** Susanne Röhr, Franziska U Jung, Alexander Pabst, Thomas Grochtdreis, Judith Dams, Michaela Nagl, Anna Renner, Rahel Hoffmann, Hans-Helmut König, Anette Kersting, Steffi G Riedel-Heller

**Affiliations:** 1 Institute of Social Medicine, Occupational Health and Public Health Medical Faculty, University of Leipzig Leipzig Germany; 2 Global Brain Health Institute Trinity College Dublin Dublin Ireland; 3 Department of Health Economics and Health Services Research Hamburg Center for Health Economics University Medical Center Hamburg-Eppendorf Hamburg Germany; 4 Department of Psychosomatic Medicine and Psychotherapy University Medical Center Leipzig Leipzig Germany

**Keywords:** app, cost-utility analysis, mHealth, posttraumatic stress, PTSD, quality-adjusted life years, randomized controlled trial, refugees, stimga, Syrian refugees, usability

## Abstract

**Background:**

Syrian refugees residing in Germany often develop posttraumatic stress as a result of the Syrian civil war, their escape, and postmigration stressors. At the same time, there is a lack of adequate treatment options. The smartphone-based app Sanadak was developed to provide cognitive behavioral therapy–based self-help in the Arabic language for Syrian refugees with posttraumatic stress.

**Objective:**

The aim of this study was to evaluate the effectiveness and cost-effectiveness of the app.

**Methods:**

In a randomized controlled trial, eligible individuals were randomly allocated to the intervention group (IG; app use) or control group (CG; psychoeducational reading material). Data were collected during structured face-to-face interviews at 3 assessments (preintervention/baseline, postintervention/after 4 weeks, follow-up/after 4 months). Using adjusted mixed-effects linear regression models, changes in posttraumatic stress and secondary outcomes were investigated as intention-to-treat (ITT) and per-protocol (PP) analysis. Cost-effectiveness was evaluated based on adjusted mean total costs, quality-adjusted life years (QALYs), and cost-effectiveness acceptability curves using the net benefit approach.

**Results:**

Of 170 screened individuals (aged 18 to 65 years), 133 were eligible and randomized to the IG (n=65) and CG (n=68). Although there was a pre-post reduction in posttraumatic stress, ITT showed no significant differences between the IG and CG after 4 weeks (Posttraumatic Diagnostic Scale for DSM-5, Diff –0.90, 95% CI –0.24 to 0.47; *P*=.52) and after 4 months (Diff –0.39, 95% CI –3.24 to 2.46; *P*=.79). The same was true for PP. Regarding secondary outcomes, ITT indicated a treatment effect for self-stigma: after 4 weeks (Self-Stigma of Mental Illness Scale/SSMIS–stereotype agreement: *d*=0.86, 95% CI 0.46 to 1.25; stereotype application: *d*=0.60, 95% CI 0.22 to 0.99) and after 4 months (*d*=0.52, 95% CI 0.12 to 0.92; *d*=0.50, 95% CI 0.10 to 0.90), the IG showed significantly lower values in self-stigma than the CG. ITT showed no significant group differences in total costs and QALYs. The probability of cost-effectiveness was 81% for a willingness-to-pay of €0 per additional QALY but decreased with increasing willingness-to-pay.

**Conclusions:**

Sanadak was not more effective in reducing mild to moderate posttraumatic stress in Syrian refugees than the control condition nor was it likely to be cost-effective. Therefore, Sanadak is not suitable as a standalone treatment. However, as the app usability was very good, no harms detected, and stigma significantly reduced, Sanadak has potential as a bridging aid within a stepped and collaborative care approach.

**Trial Registration:**

German Clinical Trials Register DRKS00013782; https://www.drks.de/drks_web/navigate.do?navigationId=trial.HTML&TRIAL_ID=DRKS00013782

**International Registered Report Identifier (IRRID):**

RR2-10.1186/s12888-019-2110-y

## Introduction

According to the United Nations Refugee Agency in 2019, the unprecedented number of 26 million individuals worldwide have been seeking shelter as refugees [1]. As a result of displacement and adverse associated experiences such as torture, trauma, and loss, refugees have an increased risk of mental ill-health [2]. In Germany, Syrians who have escaped the ongoing civil war since 2010-2011 represent the largest group among refugees. Studies have shown that Syrian refugees were typically exposed to potentially traumatizing events, increasing their vulnerability to posttraumatic stress and comorbid mental health outcomes [2]. The most frequently reported disorders associated with war and escape are posttraumatic stress disorder (PTSD) and major depression, often accompanied by somatization [3]. For example, among 518 adult Syrian refugees in Germany, 75.3% reported having witnessed and/or experienced traumatic events and 11.4% had symptoms of PTSD [2]. Furthermore, moderate to severe depression was present in 14.5% and moderate to severe generalized anxiety in 13.5% of Syrian adult refugees [2].

Current guidelines on PTSD treatment by the German Association of the Scientific Medical Societies indicate that trauma adaptive psychotherapy should be offered in a timely manner [1]. However, particularly for refugees, several barriers to treatment exist, including language and cultural barriers, legal and health insurance regulations in regard to asylum, and lack of psychoeducation [2]. In addition, proactive uptake of mental health care is low among refugees [3].

Research suggests that treatment of PTSD is effective [4]; however, in the context of the high prevalence of PTSD symptomatology among refugees, health care systems in host countries may often not have enough resources to cover the need for treatment. Therefore, eHealth interventions have been suggested as a means to close the treatment gap [5]. Moreover, as PTSD is associated with high costs of outpatient treatment, nonphysician outpatient contacts, and psychiatric contacts [6], app-based interventions could be a cost-effective alternative. Therefore, Sanadak, a smartphone-based interactive low-threshold self-help app in the Arabic language, has been developed based on evidence-driven cognitive behavioral therapy for PTSD [7]. During the development of the app, typical themes and needs of refugees as well as cultural specifics were incorporated. Therefore, focus groups were conducted to assess relevant aspects (eg, concepts of disease and disease management), which have been found to be highly recommended in comparison with traditional mental health interventions [8,9]. The content of the Sanadak app is multimodal (ie, it includes psychoeducational information to increase knowledge and awareness of PTSD and related mental health issues and self-help techniques and skills training for symptom management). In addition, a short self-test on posttraumatic symptom severity was implemented to allow for automated tailored feedback regarding progress at any time. Interactive materials, such as animated videos and audios as well as games and exercises are provided to maximize usability. Further information is detailed in the study protocol [7]. To the best of our knowledge, there is currently no comparable multimodal app intervention available for this target group that has been evaluated in terms of its effectiveness and cost-effectiveness in a randomized controlled trial (RCT). The primary aim was to evaluate the app’s effectiveness in reducing posttraumatic stress symptoms, which we hypothesized to be superior to the control condition.

## Methods

### Study Context Information

The study design is detailed in the study protocol, published elsewhere [7]. The trial was registered with the German Clinical Trials Register [DRKS00013782] on July 6, 2018. Study results are reported according to the Consolidated Standards of Reporting Trials (CONSORT) statement [10]. The app was developed by frühlingsproduktionen, a creator of eHealth interventions based in Berlin, Germany, on behalf of the study principal investigator. Sanadak app versions 1.4.0 and 1.5.0 were evaluated, first released on October 29, 2018 (no major changes between versions).

### Trial Design

After screening for eligibility, study participants were randomly allocated (1:1) to the intervention group (IG) or control group (CG), which received a psychoeducational brochure. In order to test short- as well as medium-term treatment effects, 3 face-to-face interviews were scheduled with the study participants: baseline (T0: pre), immediately after the intervention (T1: post, 4 weeks after baseline), and 4 months after baseline (T2: follow-up).

### Ethics and Guidelines

The trial was approved by the ethics committee of the Medical Faculty of the University of Leipzig, Germany (ID: 111-17-ek) and adheres to the Declaration of Helsinki and the International Council for Harmonisation of Technical Requirements for Pharmaceuticals for Human Use guidelines for good clinical practice. All participants were informed about the study aims, including clarification about data protection measures and data security according to latest legal standards. Participation was only allowed after written informed consent.

### Participants

By using a multistrategic approach to recruit Syrian refugees residing in the urban areas of Leipzig, Halle/Saale, and Dresden in Germany, potential study participants were attracted. This included bilingual posters and brochures, building contact with specific multipliers and institutions, a snowball sampling approach, and use of social media and personal contacts of the native Arabic-speaking study personnel. Recruitment strategies have been described in detail elsewhere [11]. Eligibility according to prespecified inclusion and exclusion criteria was checked during a face-to-face screening. Inclusion criteria comprised being Syrian refugee residing in Germany, aged 18 to 65 years, experiencing at least one traumatic event and subsequent mild to moderate posttraumatic stress symptom severity (score of 11 to 59) on the Posttraumatic Diagnostic Scale for DSM-5 (PDS-5) [12], and owning a compatible device to use the app (Android/iOS). Exclusion criteria included posttraumatic stress symptomatology outside of the range mentioned above; severe depressive symptoms (Patient Health Questionnaire [PHQ-9] ≥20) [13]; acute suicidal tendencies (Depressive Symptom Inventory–Suicidality Subscale [DSI-SS] ≥3) [14]; current psychotherapy, psychiatric treatment, and/or psychotropic medication; or pregnancy. If individuals were not eligible due to severity of symptoms, they received psychoeducational material on mental health care and contact information of regional initiatives that offer face-to-face support. A detailed report of the recruitment and baseline characteristics has been published previously [15].

### Interventions

Participants in the IG had the opportunity to use the self-help app via person-specific log-in data (to avoid group contamination) for 4 weeks on demand. They were advised to use the app regularly and work through the modules. Participants in the CG received psychoeducational reading material in the Arabic language covering traumatization and posttraumatic stress (identical to the information delivered by the app).

### Assessments and Outcomes

In addition to sociodemographic characteristics such as age, gender, and family status, information on residence status, employment, religious beliefs, and escape-related information were collected during standardized interviews at baseline.

The primary outcome was posttraumatic stress, measured by the PDS-5 [12]. Secondary outcomes included symptoms of depression (PHQ-9) [13]; generalized anxiety (Generalized Anxiety Disorder [GAD-7]) [16]; somatic symptoms (Physical Health Questionnaire [PHQ-15]) [17,18]; general self-efficacy (GSE) [19]; self-stigma (Self-Stigma of Mental Illness Scale–Short Form [SSMIS-SF], stereotype awareness [SSMIS-AW], stereotype agreement [SSMIS-AG], stereotype application [SSMIS-AP], and harm to self-esteem [SSMIS-HS]) [20]; resilience (Resilience Scale [RS-13]) [21]; social isolation (short form of the Lubben Social Network Scale [LSNS-6]) [22]; social support (ENRICHD Social Support Inventory [ESSI]) [23]; health-related quality of life (EuroQoL 5-Dimension 5-Level [EQ-5D-5L] and visual analog scale [EQ-VAS]) [24,25]; and posttraumatic growth (Posttraumatic Growth Inventory [PGI]) [26]. If not available, instruments were translated from German into the Arabic language using the Translation, Review, Adjudication, Pretesting, and Documentation procedure [27] involving native Arabic-speaking experts. All outcomes were assessed at T0, T1, and T2.

For the cost-effectiveness analysis, health service utilization was assessed retrospectively over 4 months at T0 and T2, using an adapted and shortened version of the German Client Socio-Demographic and Service Receipt Inventory [28]. The questionnaire covered inpatient care, rehabilitation, and outpatient physician and nonphysician services. Costs were calculated from a health care payer perspective in euros (€) for the year 2019. Monetary valuation of used health care services was conducted using standardized unit costs within the German health care system [29]. Intervention costs consisted of the costs for technical support for the Sanadak app during follow-up. Health effects were quantified by quality-adjusted life years (QALYs) observed during the 4-month follow-up period calculated by linearly interpolating EQ-5D-5L index scores from baseline to follow-up [25,30].

In the IG, we furthermore assessed information on app usability (System Usability Scale [SUS] [31]). Furthermore, deidentified metadata of the app use (ie, duration of app use during evaluation period, in minutes) stored in the app’s log files were collected.

Last, a standardized assessment at T1 and T2 was implemented to monitor potential harms due to trial participation. Harms/negative effects were defined as adverse events (AE) and severe adverse events (SAE). AE comprised an increase in target symptoms (posttraumatic and depressive symptomatology, suicidality), occurrence of novel psychological or physical symptoms, any negative events, all of which may or may not be associated with trial participation in both IG and CG. SAE were defined as events that require some form of high-intensity treatment (ie, deliberate self-harm, suicide attempt, life-threatening events, nonelective or extended hospitalization, an event causing chronic or severe disability) or fatality, including suicide.

### Sample Size

The sample size was calculated based on recent evaluations regarding the efficacy of telemedical-based treatment of PTSD symptoms [32]. Given a moderate between-group effect at follow-up 1 (Cohen *d*=0.5), a significance level of α=.05 (1-sided), and a statistical power of 1–β=0.80, optimal sample size for estimating a significant treatment effect is n=102. Considering attrition due to different circumstances (eg, change in residence status, trial termination by the participant), a dropout rate of approximately 20% may be expected, suggesting a sufficient baseline sample size of n=128 participants.

### Randomization and Masking

After participants were found to be eligible for participation, they were randomly assigned to IG or CG using a 1:1 ratio using randomized permuted blocks of 6, stratified by age and sex, which ensured both balance in sample size across groups and control of important covariates. An external, independent statistician generated the randomization block lists with a respective computer program (blockrand package written for R [R Foundation for Statistical Computing]).

The study coordinator (SR), responsible for individual group allocation, remained blind to the randomization list strata identity. Moreover, the data analyst (AP), who conducted the primary analysis concerning the hypothesized group differences (IG vs CG) in primary and secondary outcome measures (see above), was blind to group assignment.

### Data Management, Data Protection, and Quality Control

With regard to interview assessments, deidentified data entry took place immediately after each interview using the statistical software SPSS Statistics version 24 (IBM Corp), generating a password-protected and locally stored file with access granted only to study personnel who had signed data protection wavers. Concurrently, data completeness and consistency checks were conducted to ensure data integrity. With regard to data on app use, anonymous log-file data were collected during participant interaction with the app, stored at the app developer’s secured server, and collectively transferred upon completion of all intervention periods to the study investigator using Secure Sockets Layer technology to ensure data encryption and protection. In fact, specific arrangements regarding data protection (eg, no real names for program log-in) were prespecified in a data protection concept. The data protection concept detailed data handling of all collected data (ie, interview data at screening, T0, T1, and T2 and metadata of the app use) with the purpose to ensure compliance with the General Data Protection Regulation (GDPR) of the European Union. The GDPR specifies the lawful processing of personal data. The data protection concept was composed with and approved by an external lawyer specializing in data protection.

As an additional measure of data quality, auditing took place in the form of independent source data verification, performed by commissioned external statisticians. Specifically, 5% of the questionnaires at T0, T1, and T2 (source data) were randomly drawn and inspected regarding their degree of matching with the electronic data file.

### Statistical Analysis

Quality checks on baseline data revealed missing information on both outcome variables and covariates. Frequency of missing values was low (ie, 5/133 cases or less) for all variables but high for education (24/133 cases) and summed up to 27.8% for the set of baseline characteristics. Since sensitivity analysis showed that missing values were not completely at random, a complete case analysis was inappropriate. Therefore, we multiple-imputed missing baseline data using the algorithm of chained equations implemented in Stata (StataCorp LLC) with all sociodemographic variables and baseline assessments of outcome variables as predictors. The resulting pooled estimates of 25 imputed datasets were used for all analyses.

Primary analysis of trial data was intention-to-treat (ITT) as outlined in the guidelines of the CONSORT statement and its supplement for reporting eHealth trials [33]. In order to evaluate treatment effect, multilevel mixed-effects linear regression models were used, since all outcomes were approximately normally distributed. The models included an indicator of treatment group, time, and an interaction between treatment group and time as fixed effects and comprised a random intercept to control for potential heterogeneity within participants over time. All models were further adjusted for the baseline outcome score, as well as for age, gender, education (low/middle/high according to the Comparative Analysis of Social Mobility in Industrial Nations classification of education [34]), marital status, living situation, residence permit, employment, income, religious beliefs, and duration of residence in Germany as these covariates were considered to be prognostic in relation to the outcomes.

For sensitivity analysis, we performed a per-protocol (PP) analysis by excluding participants who had not used the intervention as indicated by deidentified log-in data. Mixed-effects linear regression models for both primary and secondary outcomes were also used on that restricted sample following the same procedure outlined above. The above described analyses were repeated with regard to subgroups (age: 18 to 29 years, 30 to 39 years, 40+ years; gender: male, female; level of education: low, medium, high; frequency of app use: 1 to 42.5 minutes, 42.5+ minutes; posttraumatic stress symptom severity: low, moderate).

To analyze the cost-effectiveness of Sanadak compared with receiving psychoeducational reading material on trauma and PTSD only, adjusted total costs, QALYs, and differences in total costs and QALYs between the IG and CG were calculated using mixed-effects linear regression models with robust standard errors adjusted for age, sex, costs, EQ-5D-5L indices, and PDS-5 indices at baseline. Furthermore, cost-effectiveness acceptability curves of Sanadak based on the net benefit approach were calculated accordingly by multilevel mixed-effects linear regressions based on the net monetary benefit for different willingness-to pay (WTP) thresholds [35].

Descriptive data are presented as number of observations with percentages or means with corresponding standard deviations or 95% confidence intervals. Results of the mixed-effects regression models are presented as adjusted mean differences and 95% confidence intervals in primary and secondary outcome scores between the treatment groups at both follow-ups. We also report standard effect sizes of treatment on all study outcomes at follow-up (Cohen *d*) using adjusted mean scores from the mixed models. All analyses were performed using the Stata 16.1 SE software package (StataCorp LLC). A *P* value of <.05 was considered statistically significant.

## Results

### Participants

**Figure 1:**
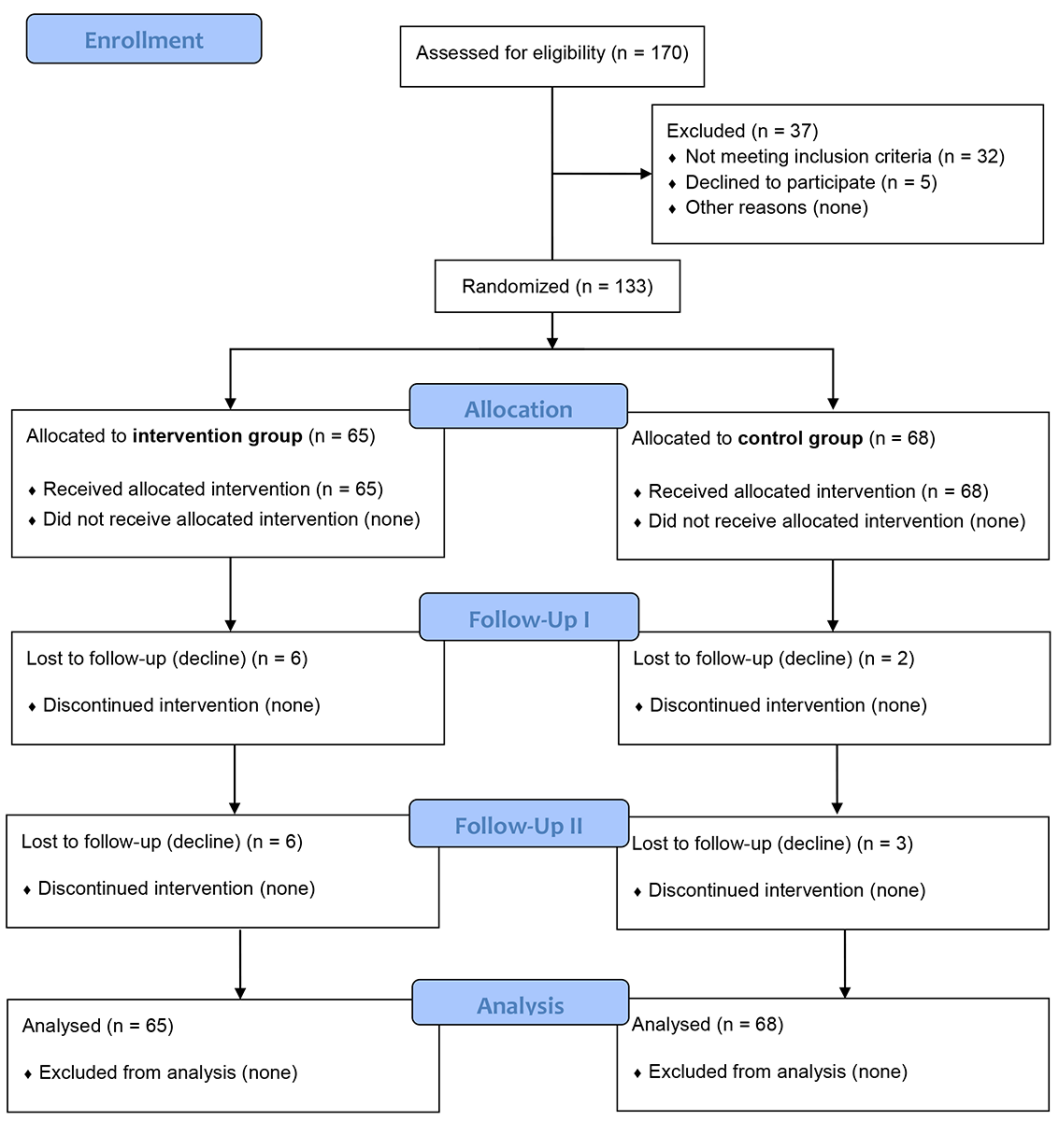
Flowchart of sample selection and attrition in the Sanadak trial.

Participants were recruited between October 2018 and December 2019, and follow-up was completed in April 2020. Figure 1 shows the flow of participants through the trial. Altogether, 170 individuals were assessed for eligibility, of which 37 were excluded as they did not meet inclusion criteria (n=32, 86.5%) or as they declined further participation (n=5, 13.5%). The eligible 133 individuals were randomly allocated either to the IG or CG. Table 1 shows baseline characteristics of the participants. The IG sample consisted of 65 participants with a mean age of 33.0 (SD 11.0, range 18 to 65) years; 66.2% (43/65) were male. The average age of the control group (n=68) was 33.7 (SD 11.4, range 18 to 65) years; 57.4% (39/68) were male. No significant differences were found for key sociodemographic variables or other baseline variables between the two groups, except for higher unemployment in the CG, indicating that the randomization was successful.

**Table 1:** Baseline characteristics of study participants of the Sanadak trial (n=133).

Characteristics		CG^a^ (n=68)	IG^b^ (n=65)
Male		39 (57.4)	43 (66.2)
Age in years, mean (SD)		33.67 (11.4)	32.98 (11.0)
**Marital status, n (%)**			
	Married	30 (44.1)	21 (32.3)
	Unmarried/divorced/widowed	38 (55.9)	44 (67.7)
**Living situation, n (%)**			
	Alone	15 (22.1)	18 (27.7)
	With family	38 (55.9)	35 (53.9)
	With others	15 (22.1)	12 (18.5)
**Education, n (%)**			
	Low	14 (20.6)	17 (26.2)
	Medium	30 (44.1)	21 (32.3)
	High	24 (35.3)	27 (41.5)
**Employment, n (%)**			
	Employed	13 (19.1)	23 (35.4)
	Unemployed	55 (80.9)	42 (64.6)
**Income per month (€), n (%)**			
	<500	13 (19.1)	14 (21.5)
	500-1000	40 (58.8)	33 (50.8)
	>1000	15 (22.2)	18 (27.7)
**Residence permit, n (%)**			
	Entitlement to political asylum	39 (57.4)	35 (53.9)
	Refugee protection	16 (23.5)	12 (18.5)
	Other (subsidiary/visa/reunification)	13 (19.1)	18 (27.7)
Religious beliefs, mean (SD)		15.01 (5.7)	14.95 (4.9)
Duration of residence in years, mean (SD)		3.53 (1.3)	3.49 (1.1)

^a^CG: control group.

^b^IG: intervention group.

### Attrition

A total of 6.0% (8/133) of the participants were lost to follow-up at the end of 4 weeks (T1). In all cases, participants refused further participation. Another 6.8% (9/133) of the sample did not complete assessments at T2. Two of those refused further participation; in 7 cases, repeated contact attempts failed. Total attrition was 12.8% (17/133). There were no significant differences between participants that did and did not complete any of the follow-up interviews nor were there significant differences between conditions in participating at T1 and T2.

### Effectiveness of the Intervention

**Figure 2:**
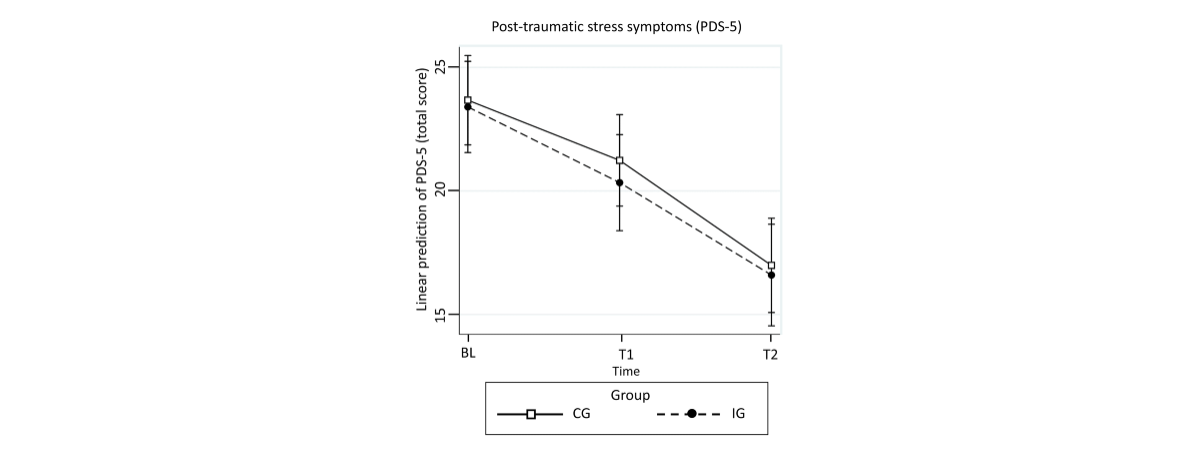
Adjusted predictions of primary outcome of posttraumatic stress symptoms (total score of the Posttraumatic Diagnostic Scale for DSM-5) at baseline and 4 weeks (T1) and 4 months (T2) after baseline in the intervention group and control group in the Sanadak trial, an Arabic language self-help app for Syrian refugees with posttraumatic stress, using mixed-effects linear regression models.

**Figure 3:**
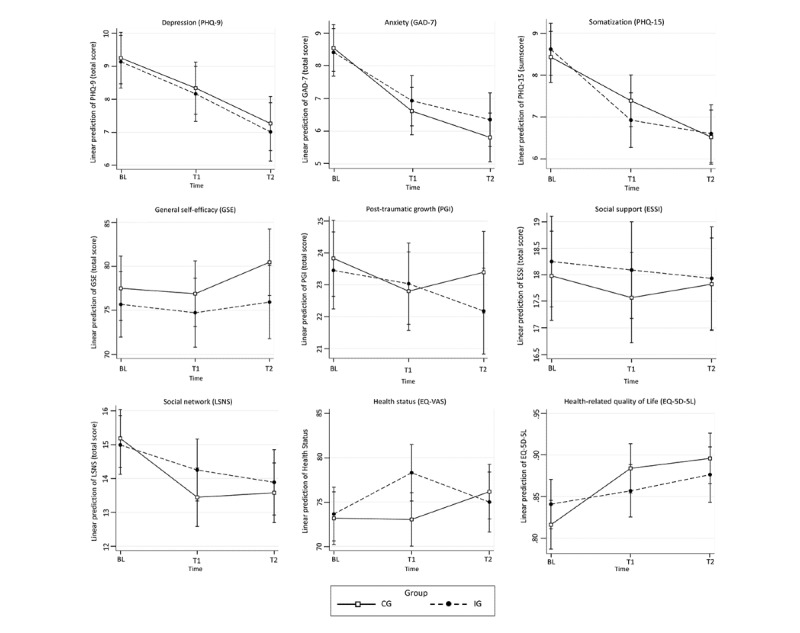
Adjusted predictions of secondary outcomes at baseline and 4 weeks (T1) and 4 months (T2) after baseline in the intervention group and control group in the Sanadak trial, an Arabic language self-help app for Syrian refugees with posttraumatic stress, using mixed-effects linear regression models. EQ-5D-5L: EuroQoL 5-Dimensions Questionnaire; EQ-VAS: EuroQoL Visual Analog Scale; ESSI: ENRICHD Social Support Inventory; GAD: General Anxiety Disorder; GSE: General Self-Efficacy; LSNS: Lubben Social Network Scale; PGI: Posttraumatic Growth Inventory; PHQ-9/-15: Patient Health Questionnaire.

**Figure 4:**
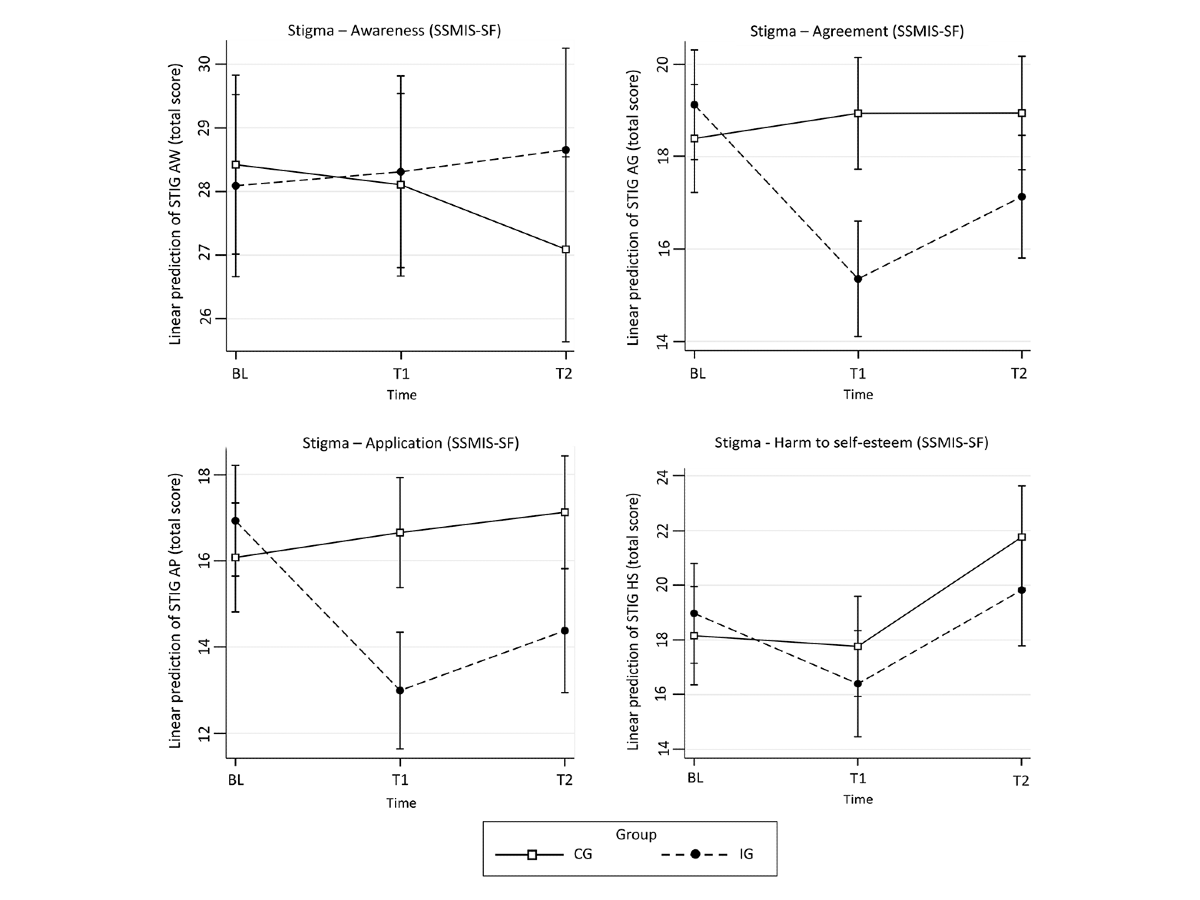
Adjusted predictions of measures of self-stigma (Self-Stigma of Mental Illness Scale–Short Form; AW: stereotype awareness, AG: stereotype agreement, AP: stereotype application, HS: harm to self-esteem) at baseline and 4 weeks (T1) and 4 months (T2) after baseline in the intervention group and control group in the Sanadak trial, an Arabic language self-help app for Syrian refugees with posttraumatic stress, using mixed-effects linear regression models.

Table 2 shows average scores for the primary (PDS-5) and secondary outcome variables (PHQ-9, GAD-7, PHQ-15, EQ-5D-5L, EQ-VAS, GSE, SSMIS-AW, SSMIS-AG, SSMIS-AP, SSMIS-HS, LNSN, ESSI, and PGI) at baseline and follow-up. Table 3 shows results of the analysis of the ITT sample for all outcomes. There were no significant differences in PTSD symptoms between the IG and CG after 4 weeks (PDS-5, Diff –0.90, 95% CI –0.24 to 0.47; *P*=.52) and after 4 months (PDS-5, Diff –0.39, 95% CI –3.24 to 2.46; *P*=.79; Figure 2). The same was true for secondary outcomes except self-stigma (Figure 3). Table 4 shows results of the PP analysis for all outcomes that scarcely differed from those in Table 3. Again, significant group differences in PTSD symptoms between the two groups were found neither after 4 weeks (PDS-5, Diff 0.10, 95% CI –2.77 to 2.98; *P*=.95) nor after 4 months (PDS-5, Diff 0.56, 95% CI –3.56 to 2.45; *P*=.72). A significant difference was found for self-stigma (SSMIS-AG and SSMIS-AP). After 4 weeks (SSMIS-AG, *d*=0.86, 95% CI 0.46 to 1.25; SSMIS-AP, *d*=0.60, 95% CI 0.22 to 0.99) and after 4 months (SSMIS-AG, *d*=0.52, 95% CI 0.12 to 0.92; SSMIS-AP, *d*=0.50, 95% CI 0.10 to 0.90), the IG showed significantly lower values in self-stigma than the CG (Figure 4).

**Table 2:** Average scores of primary and secondary outcome variables at baseline and follow-up.

Outcome			CG^a^ (n=68)						IG^b^ (n=65)					
			n		Mean		95% CI		n		Mean		95% CI	
**Primary outcome**														
	**PDS-5^c^**													
		Baseline		68		24.43		21.43-27.42		65		23.18		20.51-25.86
		4 weeks		65		21.75		18.48-25.03		59		19.66		16.61-22.72
		4 months		61		17.15		14.43-19.87		53		15.79		12.91-18.68
**Secondary outcome**														
	**PHQ-9^d^**													
		Baseline		68		9.34		7.96-10.71		65		9.15		7.97-10.34
		4 weeks		66		8.52		7.21-9.82		59		7.90		6.69-9.11
		4 months		61		7.41		4.91-7.38		53		6.79		5.48-8.11
	**GAD-7^e^**													
		Baseline		68		8.84		7.50-10.17		65		8.23		7.15-9.31
		4 weeks		66		6.98		5.73-8.24		59		6.56		5.43-7.69
		4 months		62		6.15		4.91-7.38		52		5.77		4.74-6.80
	**PHQ-15^f^**													
		Baseline		68		8.54		7.31-9.78		65		8.80		7.49-10.11
		4 weeks		66		7.44		6.22-8.66		59		6.93		5.71-8.15
		4 months		60		6.37		5.13-7.60		53		6.30		4.93-7.67
	**ED-5D-5L^g^**													
		Baseline		68		0.80		0.74-0.85		65		0.86		0.82-0.89
		4 weeks		66		0.86		0.82-0.91		59		0.88		0.85-0.92
		4 months		63		0.88		0.83-0.92		53		0.90		0.88-0.93
	**Health status**													
		Baseline		68		72.53		67.47-77.59		65		74.23		70.03-78.43
		4 weeks		66		72.98		68.85-77.12		59		78.53		74.74-82.31
		4 months		63		75.92		71.47-80.37		53		74.77		70.58-78.96
	**GSE^h^**													
		Baseline		68		28.01		27.04-28.99		65		26.78		25.49-28.08
		4 weeks		65		27.88		26.78-28.97		59		26.39		25.05-27.73
		4 months		63		28.57		27.49-29.66		53		26.45		25.25-27.66
	**SSMIS-AW^i^**													
		Baseline		68		28.63		26.62-30.65		65		27.82		26.18-29.45
		4 weeks		65		28.49		26.71-30.28		59		27.88		25.78-29.98
		4 months		63		27.43		25.61-29.25		53		28.09		26.13-30.06
	**SSMIS-AG^j^**													
		Baseline		68		17.69		16.12-19.26		65		20.05		18.37-21.72
		4 weeks		64		17.89		16.36-19.42		59		16.32		14.68-17.97
		4 months		62		17.92		16.18-19.66		53		18.23		16.46-19.99
	**SSMIS-AP^k^**													
		Baseline		68		15.31		13.82-16.80		65		17.85		16.16-19.53
		4 weeks		66		15.73		13.90-17.56		59		13.95		12.40-15.50
		4 months		63		16.13		14.40-17.85		53		15.70		13.73-17.67
	**SSMIS-HS^l^**													
		Baseline		68		18.19		15.88-20.51		65		19.22		16.83-21.60
		4 weeks		65		17.65		15.29-20.01		58		16.40		14.26-18.53
		4 months		62		21.50		18.99-24.01		53		20.3		18.32-22.29
	**LSNS-6^m^**													
		Baseline		68		15.21		13.96-16.46		65		14.95		13.60-16.31
		4 weeks		66		13.38		12.16-14.60		59		14.14		12.72-15.56
		4 months		63		13.56		12.45-14.67		53		14.13		12.83-15.44
	**ESSI^n^**													
		Baseline		68		17.66		16.39-18.93		65		18.37		17.35-19.38
		4 weeks		66		17.20		15.91-18.48		58		18.43		17.09-19.77
		4 months		63		17.52		16.40-18.65		52		18.65		17.44-19.87
	**PGI^o^**													
		Baseline		68		24.94		23.40-26.48		65		22.75		21.10-24.40
		4 weeks		65		23.69		21.87-25.52		59		22.07		19.85-24.29
		4 months		61		24.34		22.38-26.31		53		20.89		18.99-22.79
**Total costs**														
		Baseline		68		507.95		159.21-856.69		65		349.46		224.10-474.82
		4 months		57		551.85		255.59-848.11		50		306.88		201.76-412.01

^a^CG: control group.

^b^IG: intervention group.

^c^PDS-5: Posttraumatic Diagnostic Scale for DSM-5.

^d^PHQ-9: Patient Health Questionnaire.

^e^GAD-7: Generalized Anxiety Disorder.

^f^PHQ-15: Physical Health Questionnaire.

^g^ED-5D-5L: EuroQoL 5-Dimension 5-Level.

^h^GSE: General Self-Efficacy.

^i^SSMIS-AW: Self-Stigma of Mental Illness Scale–stereotype awareness.

^j^SSMIS-AG: Self-Stigma of Mental Illness Scale–stereotype agreement.

^k^SSMIS-AP: Self-Stigma of Mental Illness Scale–stereotype application.

^l^SSMIS-HS: Self-Stigma of Mental Illness Scale–harm to self-esteem.

^m^LSNS-6: Lubben Social Network Scale.

^n^ESSI: ENRICHD Social Support Inventory.

^o^PGI: Posttraumatic Growth Inventory.

**Table 3:** Results of the Sanadak trial based on intention-to-treat analysis.

Outcome			n		Diff^a^		95% CI		*P* value		*d*		95% CI	
**Primary outcome**														
	**PDS-5^b^**													
		Baseline		—		—		—		—		—		—
		4 weeks		124		–0.901		–3.624 to 1.823		.52		0.118		–0.235 to 0.471
		4 months		114		–0.390		–3.238 to 2.457		.79		0.051		–0.317 to 0.419
**Secondary outcome**														
	**PHQ-9^c^**													
		Baseline		—		—		—		—		—		—
		4 weeks		125		–0.176		–1.343 to 0.991		.77		0.054		–0.298 to 0.405
		4 months		114		–0.255		–1.480 to 0.970		.68		0.078		–0.291 to 0.446
	**GAD-7^d^**													
		Baseline		—		—		—		—		—		—
		4 weeks		125		0.317		–0.752 to 1.386		.56		–0.106		–0.458 to 0.246
		4 months		114		0.541		–0.582 to 1.664		.35		–0.180		–0.549 to 0.190
	**PHQ-15^e^**													
		Baseline		—		—		—		—		—		—
		4 weeks		125		–0.463		–1.373 to 0.447		.32		0.181		–0.171 to 0.533
		4 months		113		0.080		–0.879 to 1.039		.87		–0.031		–0.401 to 0.338
	**ED-5D-5L^f^**													
		Baseline		—		—		—		—		—		—
		4 weeks		125		–0.027		–0.071 to 0.017		.23		0.219		–0.133 to 0.571
		4 months		116		–0.019		–0.065 to 0.026		.40		0.158		–0.208 to 0.524
	**Health status**													
		Baseline		—		—		—		—		—		—
		4 weeks		125		5.254		0.830 to 9.679		.02		–0.421		–0.776 to –0.065
		4 months		116		–1.165		–5.783 to 3.452		.62		0.093		–0.272 to 0.459
	**GSE^g^**													
		Baseline		—		—		—		—		—		—
		4 weeks		124		5.254		0.830 to 9.679		.44		0.140		–0.213 to 0.493
		4 months		116		–0.905		–2.043 to 0.233		.12		0.295		–0.073 to 0.661
	**SSMIS-AW^h^**													
		Baseline		—		—		—		—		—		—
		4 weeks		124		0.203		–1.901 to 2.308		.85		–0.034		–0.387 to 0.318
		4 months		116		1.563		0.624 to 3.749		.16		–0.265		0.631 to 0.103
	**SSMIS-AG^i^**													
		Baseline		—		—		—		—		—		—
		4 weeks		122		–3.587		–5.355 to –1.819		<.001		0.732		0.364 to 1.097
		4 months		114		–1.813		–3.655 to 0.028		.05		0.369		–0.003 to 0.739
	**SSMIS-AP^j^**													
		Baseline		—		—		—		—		—		—
		4 weeks		125		–3.658		–5.543 to –1.773		<.001		0.691		0.329 to 1.052
		4 months		116		–2.743		–4.712 to 0.774		.01		0.517		0.145 to 0.888
	**SSMIS-HS^k^**													
		Baseline		—		—		—		—		—		—
		4 weeks		123		–1.370		–4.069 to 1.329		.32		0.182		–0.173 to 0.536
		4 months		115		–1.938		–4.739 to 0.863		.18		0.257		–0.112 to 0.625
	**LSNS-6^l^**													
		Baseline		—		—		—		—		—		—
		4 weeks		125		0.804		–0.471 to 2.079		.22		–0.224		–0.576 to 0.128
		4 months		116		0.310		–1.016 to 1.636		.65		–0.087		–0.452 to 0.279
	**ESSI^m^**													
		Baseline		—		—		—		—		—		—
		4 weeks		124		0.520		–0.741 to 1.781		.42		–0.147		–0.500 to 0.206
		4 months		115		0.110		–1.213 to 1.432		.87		–0.031		–0.398 to 0.336
	**PGI^n^**													
		Baseline		—		—		—		—		—		—
		4 weeks		122		0.233		–1.517 to 2.037		.80		–0.047		–0.402 to 0.309
		4 months		111		–1.217		–3.110 to 0.676		.21		0.243		–0.131 to 0.617

^a^Diff: difference.

^b^PDS-5: Posttraumatic Diagnostic Scale for DSM-5.

^c^PHQ-9: Patient Health Questionnaire.

^d^GAD-7: Generalized Anxiety Disorder.

^e^PHQ-15: Physical Health Questionnaire.

^f^ED-5D-5L: EuroQoL 5-Dimension 5-Level.

^g^GSE: General Self-Efficacy.

^h^SSMIS-AW: Self-Stigma of Mental Illness Scale–stereotype awareness.

^i^SSMIS-AG: Self-Stigma of Mental Illness Scale–stereotype agreement.

^j^SSMIS-AP: Self-Stigma of Mental Illness Scale–stereotype application.

^k^SSMIS-HS: Self-Stigma of Mental Illness Scale–harm to self-esteem.

^l^LSNS-6: Lubben Social Network Scale.

^m^ESSI: ENRICHD Social Support Inventory.

^n^PGI: Posttraumatic Growth Inventory.

**Table 4:** Results of the Sanadak trial based on per-protocol analysis.

Outcome				n		Diff^a^		95% CI		*P* value		*d*		95% CI
**Primary outcome**														
	**PDS-5^b^**													
		Baseline	—		—		—		—		—		—	
		4 weeks	111		0.102		–2.774 to 2.978		.95		–0.010		–0.391 to 0.364	
		4 months	102		0.556		–3.560 to 2.448		.72		0.074		–0.322 to 0.470	
**Secondary outcome**														
	**PHQ-9^c^**													
		Baseline	—		—		—		—		—		—	
		4 weeks	112		–0.194		–1.450 to 1.062		.76		0.059		–0.318 to 0.435	
		4 months	102		–0.503		–1.822 to 0.816		.46		0.153		–0.243 to 0.549	
	**GAD-7^d^**													
		Baseline	—		—		—		—		—		—	
		4 weeks	112		0.377		–0.800 to 1.554		.53		–0.120		–0.499 to 0.254	
		4 months	102		0.822		–0.417 to 2.061		.19		–0.270		–0.667 to 0.131	
	**PHQ-15^e^**													
		Baseline	—		—		—		—		—		—	
		4 weeks	112		–0.04		–1.027 to 0.947		.94		0.015		–0.361 to 0.392	
		4 months	101		0.424		–0.616 to 1.465		.42		–0.170		–0.562 to 0.234	
	**ED-5D-5L^f^**													
		Baseline	—		—		—		—		—		—	
		4 weeks	112		–0.024		–0.073 to 0.025		.34		0.189		–0.189 to 0.556	
		4 months	104		–0.021		–0.071 to 0.030		.43		0.163		–0.163 to 0.556	
	**Health status**													
		Baseline	—		—		—		—		—		—	
		4 weeks	112		4.464		–0.421 to 9.350		.07		–0.350		–0.727 to 0.032	
		4 months	104		–0.228		–5.338 to 4.881		.93		0.017		–0.376 to 0.411	
	**GSE^g^**													
		Baseline	—		—		—		—		—		—	
		4 weeks	111		–0.581		–1.733 to 0.572		.32		0.194		–0.185 to 0.572	
		4 months	104		–0.862		–2.060 to 0.336		.16		0.288		–0.108 to 0.682	
	**SSMIS-AW^h^**													
		Baseline	—		—		—		—		—		—	
		4 weeks	111		0.158		–2.088 to 2.405		.89		–0.030		–0.405 to 0.351	
		4 months	104		0.635		–1.704 to 2.974		.60		–0.110		–0.502 to 0.286	
	**SSMIS-AG^i^**													
		Baseline	—		—		—		—		—		—	
		4 weeks	109		–3.996		–5.795 to –2.197		<.001		0.858		0.460 to 1.254	
		4 months	102		–2.435		–4.313 to –0.557		.01		0.522		0.119 to 0.923	
	**SSMIS-AP^j^**													
		Baseline	—		—		—		—		—		—	
		4 weeks	112		–3.201		–5.245 to –1.158		.01		0.601		0.215 to 0.985	
		4 months	104		–2.649		–4.787 to –0.510		.02		0.497		0.097 to 0.895	
	**SSMIS-HS^k^**													
		Baseline	—		—		—		—		—		—	
		4 weeks	110		–0.446		–3.391 to 2.500		.77		0.058		–0.322 to 0.438	
		4 months	103		–1.877		–4.935 to 1.181		.23		0.246		–0.151 to 0.641	
	**LSNS-6^l^**													
		Baseline	—		—		—		—		—		—	
		4 weeks	112		0.864		–0.509 to 2.233		.22		–0.240		–0.618 to 0.137	
		4 months	104		0.642		–0.785 to 2.070		.38		–0.180		–0.574 to 0.214	
	**ESSI^m^**													
		Baseline	—		—		—		—		—		—	
		4 weeks	111		0.783		–0.613 to 2.179		.27		–0.220		–0.595 to 0.165	
		4 months	103		0.059		–1.411 to 1.528		.94		–0.020		–0.412 to 0.380	
	**PGI^n^**													
		Baseline	—		—		—		—		—		—	
		4 weeks	109		0.46		–1.455 to 2.374		.64		–0.090		–0.473 to 0.287	
		4 months	99		–1.455 to 2.374		–2.090 to 1.927		.94		0.017		–0.383 to 0.416	

^a^Diff: difference.

^b^PDS-5: Posttraumatic Diagnostic Scale for DSM-5.

^c^PHQ-9: Patient Health Questionnaire.

^d^GAD-7: Generalized Anxiety Disorder.

^e^PHQ-15: Physical Health Questionnaire.

^f^ED-5D-5L: EuroQoL 5-Dimension 5-Level.

^g^GSE: General Self-Efficacy.

^h^SSMIS-AW: Self-Stigma of Mental Illness Scale–stereotype awareness.

^i^SSMIS-AG: Self-Stigma of Mental Illness Scale–stereotype agreement.

^j^SSMIS-AP: Self-Stigma of Mental Illness Scale–stereotype application.

^k^SSMIS-HS: Self-Stigma of Mental Illness Scale–harm to self-esteem.

^l^LSNS-6: Lubben Social Network Scale.

^m^ESSI: ENRICHD Social Support Inventory.

^n^PGI: Posttraumatic Growth Inventory.

### Ancillary Analyses

None of the subgroup analyses in regard to age groups, gender, education, app use frequency, and posttraumatic stress symptom severity indicated any significant effect apart from reduced self-stigma as seen in the main analysis (results not further shown).

### Cost-Effectiveness of the Intervention

**Figure 5:**
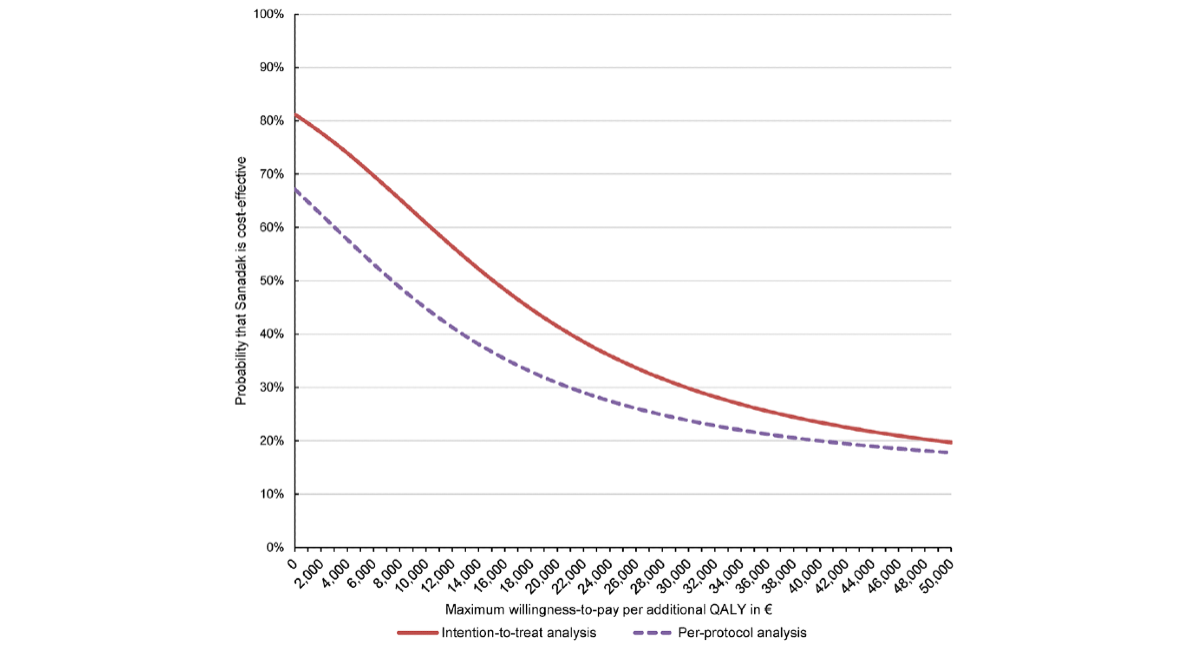
Adjusted cost-effectiveness acceptability curves for an additional quality-adjusted life year based on intention-to-treat analysis and per-protocol analysis (adjusted for age, sex, costs, EQ-5D-5L index, and PDS-5 index at baseline by multilevel mixed-effects linear regression with robust standard errors with research sites as random effect). QALY: quality-adjusted life year.

During the 4-month follow-up, mean adjusted total costs were €384 in the IG (including €52 intervention costs) and €484 in the CG (Table 5), with the difference not being statistically significant in the ITT analysis (−€100, *P*=.38). Mean adjusted QALYs were 0.29 in the IG and 0.29 in the CG, with the difference not being statistically significant (−.004, *P*=.35; Table 5). In the PP analysis, the differences in mean adjusted total costs (−€52, *P*=.11) and in mean adjusted QALYs (−.005, *P*=.34) between the IG and CG were not statistically significant. The probability for cost-effectiveness at a WTP of €0 per additional QALY was 81% (67%) in the intention-to-treat analysis (per protocol analysis). For a WTP of €50.000 per additional QALY, the probability for cost-effectiveness was 20% (18%; Figure 5).

**Table 5:** Adjusted mean costs (by cost category) and quality-adjusted life years (QALYs), and differences between intervention group and control group in mean costs (by cost category) and QALYs during 4-month follow-up based on intention-to-treat analysis and per-protocol analysis^a^.

Cost category/measure of health effect		N	IG^b^, mean (SE^c^)	CG^d^, mean (SE)	Diff^e^ IG–CG	*P* value
**ITT^f^ ($)**						
	Inpatient care and rehabilitation	116	148 (52)	250 (93)	−103 (87)	.24
	Outpatient physician services	108	180 (31)	214 (36)	−33 (46)	.47
	Outpatient nonphysician services	114	13 (7)	20 (7)	−7 (10)	.50
	Total costs	107	384 (67)	484 (111)	−100 (112)	.38
	QALYs^g^	116	0.290 (0.004)	0.294 (0.004)	−0.004 (0.005)	.35
**PP^h^ ($)**						
	Inpatient care and rehabilitation	104	180 (64)	258 (93)	−78 (88)	.38
	Outpatient physician services	96	195 (40)	205 (34)	−10 (50)	.84
	Outpatient nonphysician services	102	19 (10)	18 (6)	0 (11)	.98
	Total costs	95	433 (83)	484 (111)	−52 (117)	.66
	QALYs	104	0.288 (0.004)	0.293 (0.004)	−0.005 (0.005)	.34

^a^Adjusted for age, sex, costs, EQ-5D-5L index and PDS-5 index at baseline using mixed-effects linear regression models with robust standard errors.

^b^IG: intervention group.

^c^SE: standard error.

^d^CG: control group.

^e^Diff: difference.

^f^ITT: intention to treat.

^g^QALY: quality-adjusted life year.

^h^PP: per protocol.

### App Usability

At T1, participants in the IG were asked to rate the usability (SUS) of the app; 89.2% (58/65) of participants completed the SUS, indicating a mean usability score of 78.9 (SD 12.6; range 50.0 to 97.5; possible scoring range 0 to 100).

### Harms

Across the intervention and follow-up period, one AE occurred with relation to the trial participation. The participant self-reported increased anxiety at T1 which, however, was not quantified by the GAD-7 scores comparing baseline and T1. The participant wanted to remain in the trial. Follow-up at T2 showed that the AE had resolved. No SAE occurred.

### Source Data Verification

Across all assessment waves, 36 paper-and-pencil questionnaires (source data) were randomly drawn and compared with the electronic data file. For each questionnaire, up to 16 case report forms were inspected. Altogether, 7837 items were checked and 56 errors identified. This cumulated in a total error rate of 0.71%.

## Discussion

### Principal Findings

The smartphone-based app Sanadak, a self-help intervention in the Arabic language, was primarily developed for reducing posttraumatic stress in Syrian refugees residing in Germany as there is a gap of timely and culturally appropriate treatment. Its effectiveness was evaluated in an RCT with 133 Syrian refugees aged 18 to 65 years. Although symptom severity decreased, Sanadak was not superior in reducing mild to moderate posttraumatic stress in Syrian refugees in the short-term (4 weeks) and midterm (4 months) compared with the CG, who received psychoeducational reading material on trauma and PTSD. Likewise, the app showed no effectiveness regarding secondary outcomes, including depressive symptoms, anxiety, somatization, posttraumatic growth, general self-efficacy, social support, social isolation, health-related quality of life, and health state. However, there was a significant treatment effect for aspects of self-stigma: stereotype agreement as well as stereotype application reduced significantly in the short-term and midterm in the IG compared with the CG. Moreover, Sanadak is unlikely to be cost-effective. The app showed a small but insignificant reduction in total costs compared with the CG. As the number of QALYs tended to be (insignificantly) higher in the CG than in the IG, the probability of cost-effectiveness decreased with increasing willingness to pay per additional QALY, reaching only 20% at a WTP of €50.000. In a model-based economic evaluation, self-help without support also showed a small reduction in total costs of £235 (€199) compared with no treatment and was unlikely to be cost-effective compared with other treatment options, with a probability of cost-effectiveness of only 42% for a WTP of £20.000 (€23.624) per additional QALY [36].

Although evidence-based treatments are available for mild to severe PTSD and cognitive-behavioral therapies are among the recommended treatments [37], findings on the efficacy of app-based delivered interventions remain inconclusive and rely on a small number of studies [38-40]. In fact, a recent meta-analysis that identified 5 RCTs evaluating app-based interventions for PTSD symptoms found that the use of such apps is associated with reductions in PTSD symptoms, but that there was little evidence to suggest that apps were more effective than control conditions (usually waitlist) [40]. Another recent meta-analysis with 2 RCTs and 4 pre-post studies that focused on self-help apps for subthreshold or full PTSD likewise concluded that respective apps were not more effective than waitlist controls despite positive pre-post effects [41]. This is in line with the results of our trial, although our study differed regarding the target population. While the cited studies targeted either community-dwelling individuals or military/veteran populations, we were focusing on Syrian refugees residing in Germany. We could not identify similar app-based interventions targeting (Syrian) refugees with posttraumatic stress that have been previously evaluated in an RCT. There are a few apps targeting various mental health outcomes in (Syrian) refugees. However, they have either not been evaluated (eg, ALMHAR: self-help app for psychoeducation about emotions regulation [42]) or evaluation is ongoing (Smartphone Mediated Intervention for Learning Emotional Regulation of Sadness/SMILERS: self-help app for depressive symptoms [43]; BALSAM: self-help app for psychoeducation about emotions regulation as part of the Mental health in refugees and asylum seekers/MEHIRA project [44]; Step-by-Step: self-help app for depressive symptoms [45]). Evaluation results of the latter three apps will provide further valuable information on the effectiveness of app-based delivered mental health interventions in refugees. In general, regardless of targeted groups, there is no shortage of apps aiming to address posttraumatic stress. A systematic review by Sander and colleagues [46] identified 69 apps in Google Play and the iOS App Store. The overall app quality was found to be medium, and the authors only distinguished one app (1.4%) that had been scientifically evaluated in an RCT.

The literature provides more evidence regarding the effectiveness of interventions for posttraumatic stress in refugees delivered via internet or face-to-face. For example, in a web-based psychotherapy for war-traumatized Arab patients (focused on Iraq), posttraumatic stress was significantly reduced from baseline to posttreatment in the IG compared with the CG and effects sustained at 3-month follow-up [47]. Regarding face-to-face interventions, systematic reviews and meta-analyses found cognitive behavioral therapy, eye movement desensitization and reprocessing treatment and narrative exposure therapy [48,49] as well as psychosocial interventions [50] beneficial for certain populations of refugees; however, this evidence largely stems from settings in high-income countries, with only low-quality evidence from humanitarian settings in low- and middle-income countries [51].

The question is why treatment for posttraumatic stress may be less effective if delivered via apps compared with internet-based or face-to-face treatments. Due to the small number of studies, this remains to be investigated. However, it is likely that brief intervention periods, the self-management approach without clinical expert guidance or support, and the use of self-report measures are explaining factors [40]. For example, we advised using the app as much and as regularly as possible over the 4-week intervention period; however, the logged mean time of app use was 42.5 minutes, with 22 IG participants (32.3%) having never used the app beyond the onboarding module (psychoeducation about posttraumatic stress). This requires thinking about strategies on how to increase engagement with the app, potentially using push notification reminders (not implemented in Sanadak due to data protection measures), motivational calls by study personnel, or detailed working schedules over a certain period of time on how and when to use the app. Potentially, 4 weeks is too short to obtain significant treatment effects relative to a control condition. We did not find differential results in regard to sociodemographic subgroups (age, gender, education, income, employment status), which further supports that lack of effectiveness is rather associated with the intervention delivery mode and use.

On a positive note, the SUS usability score of Sanadak was found to be above average, indicating very good usability. There were no harms associated with the use of Sanadak, and pre-post comparisons indicated a significant reduction in posttraumatic stress, even though not more than in the control condition. Moreover, Sanadak significantly reduced self-stigma regarding mental health relative to the CG. This aspect should not be underestimated. Mental health stigma has been named a key barrier to help seeking in refugees with posttraumatic stress [52]. By reducing mental health stigma, traumatized refugees in need of help may be more willing to take up respective treatment. As such, Sanadak may serve as a pathway to conventional face-to-face treatments.

### Strengths and Limitations

The study has several strengths: a robust RCT design to determine effectiveness, adequate power, midterm follow-up assessment and low attrition, monitoring for potential harms as well as rigorous data quality control and imputation strategies for missing values, which decrease risk of bias in determining trial results. There are also limitations to consider. First, the multistrategic recruitment that heavily relied on snowball sampling techniques may limit generalizability of our findings to other traumatized populations. Moreover, the control condition which saw the provision of psychoeducational reading material in the Arabic language may not have been ideal in determining the app’s treatment effect as building awareness on posttraumatic stress symptoms in the CG may been associated with symptom severity reduction. Potentially, a mere waiting list condition would have led to differential results. Moreover, assessments heavily relied on self-report assessments. There were no in-depth clinically diagnostic interviews to assess outcomes. As service use was assessed retrospectively over 4 months using an adapted and shortened version of a service receipt inventory, it is possible that health care services utilization was not completely assessed and a potentially delayed effect of Sanadak on total health care costs could not been identified.

### Conclusions

Sanadak, an interactive self-help app in the Arabic language, showed reductions in posttraumatic stress in Syrian refugees residing in Germany in a pre-post comparison but was not superior to the control condition. This is in line with similar studies evaluating apps for posttraumatic stress, although targeting different populations. In addition, Sanadak is unlikely to be cost-effective. Future studies that investigate reasons for the limited effectiveness of respective apps are warranted to allow for improved app development and delivery modes. However, there were no harms associated with the use of Sanadak, and importantly, we found a significant and sustained treatment effect for reducing self-stigma. Consequently, Sanadak cannot be recommended as a standalone treatment for posttraumatic stress in refugee populations, but trial results indicate potential usefulness as a bridging aid, particularly as the usability was found to be very good, for the uptake of more effective internet-based or face-to-face treatments within a stepped and collaborative care approach.

## Appendix

Multimedia Appendix 1 CONSORT-eHEALTH checklist (V 1.6.1).

## Abbreviations

AE: adverse events
CG: control group
CONSORT: Consolidated Standards of Reporting Trials
DSI-SS: Depressive Symptom Inventory–Suicidality Subscale
EQ-5D-5L: EuroQoL 5-Dimension 5-Level
EQ-VAS: EuroQoL visual analog scale
ESSI: ENRICHD Social Support Inventory
GAD-7: Generalized Anxiety Disorder
GDPR: General Data Protection Regulation
GSE: General Self-Efficacy
IG: intervention group
ITT: intention-to-treat
LSNS-6: Lubben Social Network Scale
PDS-5: Posttraumatic Diagnostic Scale for DSM-5
PGI: Posttraumatic Growth Inventory
PHQ-9: Patient Health Questionnaire
PHQ-15: Physical Health Questionnaire
PP: per-protocol
PTSD: posttraumatic stress disorder
QALY: quality-adjusted life year
RCT: randomized controlled trial
RS-13: Resilience Scale
SAE: severe adverse events
SSMIS-AG: stereotype agreement
SSMIS-AW: stereotype awareness
SSMIS-AP: stereotype application
SSMIS-HS: harm to self-esteem
SSMIS-SF: Self-Stigma of Mental Illness Scale–Short Form
SUS: System Usability Scale
T0: baseline
T1: 4 weeks after baseline
T2: 4 months after baseline
WTP: willingness-to pay
