# Correction: A Self-Help App for Syrian Refugees With Posttraumatic Stress (Sanadak): Randomized Controlled Trial

**DOI:** 10.2196/28336

**Published:** 2021-03-05

**Authors:** Susanne Röhr, Franziska U Jung, Alexander Pabst, Thomas Grochtdreis, Judith Dams, Michaela Nagl, Anna Renner, Rahel Hoffmann, Hans-Helmut König, Anette Kersting, Steffi G Riedel-Heller

**Affiliations:** 1 Institute of Social Medicine, Occupational Health and Public Health Medical Faculty, University of Leipzig Leipzig Germany; 2 Global Brain Health Institute Trinity College Dublin Dublin Ireland; 3 Department of Health Economics and Health Services Research Hamburg Center for Health Economics University Medical Center Hamburg-Eppendorf Hamburg Germany; 4 Department of Psychosomatic Medicine and Psychotherapy University Medical Center Leipzig Leipzig Germany

In “A Self-Help App for Syrian Refugees With Posttraumatic Stress (Sanadak): Randomized Controlled Trial” (JMIR Mhealth Uhealth 2021;9(1):e24807) one error was noted.

In Table 4, in the row “ED-5D-5L” and sub-row “4 weeks”, under the final column “95% CI”, the correct value was not rendered and appeared only as “t”. This value has been corrected to “–0.189 to 0.556”.

The correction will appear in the online version of the paper on the JMIR Publications website on March 5, 2021, together with the publication of this correction notice. Because this was made after submission to PubMed, PubMed Central, and other full-text repositories, the corrected article has also been resubmitted to those repositories.

